# Formative Research for the Development of Evidence-Based Targeted Water, Sanitation, and Hygiene Interventions to Reduce Cholera in Hotspots in the Democratic Republic of the Congo: Preventative Intervention for Cholera for 7 Days (PICHA7) Program

**DOI:** 10.3390/ijerph191912243

**Published:** 2022-09-27

**Authors:** Lucien Bisimwa, Camille Williams, Jean-Claude Bisimwa, Presence Sanvura, Kelly Endres, Elizabeth Thomas, Jamie Perin, Cirhuza Cikomola, Justin Bengehya, Ghislain Maheshe, Alain Mwishingo, Christine Marie George

**Affiliations:** 1 Department of International Health, Johns Hopkins Bloomberg School of Public Health, Baltimore, MD 21205-2103, USA; 2 Center for Tropical Diseases & Global Health, Université Catholique de Bukavu, Bukavu B.P 265, Democratic Republic of the Congo; 3 Bureau de l’Information Sanitaire, Surveillance Epidémiologique et Recherche Scientifique Division Provinciale de la Santé/Sud Kivu, Ministère de la Santé Publique, Hygiène et Prévention, Bukavu B.P 265, Democratic Republic of the Congo; 4 Faculty of Medicine, Université Catholique de Bukavu, Bukavu B.P 265, Democratic Republic of the Congo

**Keywords:** cholera, formative research, Democratic Republic of the Congo, water, sanitation, hygiene

## Abstract

Compared to the general public, household members of cholera patients are at a 100 times higher risk of contracting cholera during the 7-day high-risk period after a cholera patient has been admitted to a health facility for treatment. The Preventative-Intervention-for-Cholera-for-7-days (PICHA7) program aims to reduce household transmission of cholera during this 7-day high-risk period through a health facility-initiated water, sanitation, and hygiene (WASH) program promoting handwashing with soap, water treatment, and safe water storage. The PICHA7 program is delivered to cholera patient households through: (1) a pictorial flipbook delivered by a health promoter; (2) a cholera prevention package (handwashing station, drinking water vessel with lid and tap, and chlorine tablets); and (3) weekly WASH mobile messages sent to patient households in the Democratic Republic of the Congo (DRC). The objectives of this study were to conduct formative research to identify facilitators and barriers of the promoted WASH behaviors for cholera patient households and to tailor the PICHA7 program to target these facilitators and barriers. Formative research included 93 semi-structured interviews with diarrhea patient households and healthcare workers during exploratory research and a pilot study of 518 participants. Barriers to the promoted WASH behaviors identified during exploratory and pilot study interviews included: (1) low awareness of cholera transmission and prevention; (2) unaffordability of soap for handwashing; and (3) intermittent access to water limiting water for handwashing. For intervention development, narratives of the lived experiences of patient households in our study were presented by health promoters to describe cholera transmission and prevention, and soapy water and ash were promoted in the program flipbook and mobile messages to address the affordability of soap for handwashing. A jerry can was provided to allow for additional water storage, and a tap with a slower flow rate was attached to the handwashing station to reduce the amount of water required for handwashing. The pilot findings indicate that the PICHA7 program has high user acceptability and is feasible to deliver to cholera patients that present at health facilities for treatment in our study setting. Formative research allowed for tailoring this targeted WASH program for cholera patient households in the DRC.

## 1. Introduction

Globally, there are 2.9 million cholera cases annually in cholera-endemic countries, which result in 95,000 deaths [1]. The Democratic Republic of the Congo (DRC) accounts for an estimated 189,000 cholera cases each year, with 7100 cases resulting in death [1,2]. The majority of cholera cases are found in cholera “hotspots”, such as eastern DRC in the Great Lakes Region [3,4,5,6]. Household members of cholera patients are at a 100 times higher risk of contracting cholera in the 7-day high-risk period after a cholera patient is admitted to a health facility compared to the general population [7,8]. Water, sanitation, and hygiene (WASH) interventions, such as handwashing with soap and water treatment, have the potential to reduce cholera transmission by interrupting the fecal–oral pathway by which cholera is spread [9]. Despite this, there are few interventions that target reducing cholera transmission among household members of cholera patients [10,11]. Furthermore, there has been little formative research conducted to identify the facilitators and barriers of WASH behaviors for cholera patient households [12,13]. 

### 1.1. Cholera-Hospital-Based-Intervention-for-7-Days (CHoBI7) Program

In an effort to reduce household transmission of cholera in Bangladesh, our research group developed the health facility-initiated Cholera-Hospital-Based-Intervention-for-7-days (CHoBI7) WASH program. This program focuses on promoting handwashing with soap and water treatment to the household members of cholera patients during the 7-day high-risk period after the cholera patient presents in a health facility for treatment to reduce household transmission of cholera [8,14,15,16]. The acronym, *chobi*, means “picture” in Bangla, for the pictorial WASH modules delivered as part of the program, and “7” for the 7-day high-risk period for household cholera transmission. CHoBI7 includes: (1) a pictorial module, delivered using a flipbook, on how diarrheal diseases spread, with instructions on proper handwashing with soap, water treatment, and safe water storage (in a completely covered container); and (2) a diarrhea prevention package, including chlorine tablets for water treatment and soapy water bottles (a low-cost alternative to bar soap made using detergent and water [17]). A health promoter delivers this module and diarrhea prevention package to patients and their accompanying family members during a session in the health facility and through home visits during the 7-day high-risk period. Our initial randomized controlled trial (RCT) of the CHoBI7 program for cholera patients in urban slums of Bangladesh demonstrated this program was effective in significantly reducing cholera and promoting sustained higher handwashing with soap and improved stored drinking water quality 12 months after program delivery [18]. 

To build the evidence needed to take the CHoBI7 program to scale in Bangladesh, we partnered with the Bangladesh Ministry of Health to develop and evaluate scalable approaches for CHoBI7 delivery. The CHoBI7 mobile health (mHealth) program (weekly WASH-related voice and text messages for 12 months) was developed as a low-cost scalable approach to reinforce the CHoBI7 WASH modules delivered in the health facility, without the need for home visits [12,13]. To reach a larger number of beneficiaries, we broadened the scope of CHoBI7 to include all diarrhea patients (irrespective of etiology). In the RCT of the CHoBI7 mHealth program, it was found to significantly reduce pediatric diarrhea and improve child growth (reduced stunting) and led to sustained increases in handwashing with soap and stored drinking water quality during the 12-month program [19]. 

### 1.2. The Preventative-Intervention-for-Cholera-for-7-Days (PICHA7) Program

To expand the evidence-based research on WASH programs targeted to diarrhea patient households in a sub-Saharan African setting, we partnered with the DRC Ministry of Health to develop and evaluate the Preventative-Intervention-for-Cholera-for-7-Days (PICHA7) program, a targeted WASH program for household members of cholera and severe diarrhea patients. The acronym, *picha,* means “picture” in Swahili because of the pictorial WASH modules included in this program, and “7” for the 7-day high-risk period for the household members of diarrhea patients for subsequent diarrheal disease transmission. 

The objectives of this study were to conduct formative research to: (1) identify facilitators and barriers to handwashing with soap, water treatment, and safe water storage in eastern DRC to inform the content and implementation of the PICHA7 program; and (2) to tailor the PICHA7 program to target these facilitators and barriers through an iterative pilot study, making refinements to the intervention based on participant feedback.

## 2. Methods

### 2.1. Study Setting and Rationale

**Figure 1:**
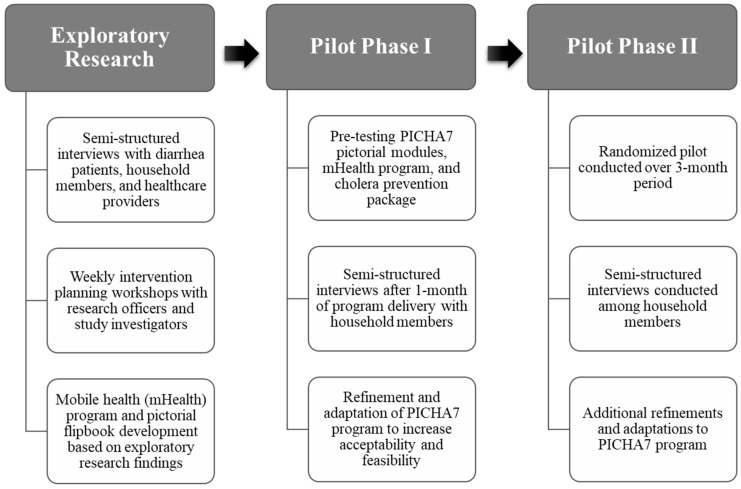
Overview of PICHA7 formative research activities.

This formative research was conducted in Bukavu, South Kivu, DRC, from February 2020 to October 2021. Bukavu is a city of >1 million people in the mountains of eastern DRC. Participants included caregivers of young children, diarrhea patients, and healthcare providers. All participants in the semi-structured interviews had to be 12 years of age or older. Formative research activities were informed by the Integrated Behavioral Model for Water, Sanitation, and Hygiene (IBM-WASH), which is based on multi-dimensional factors that influence behavior change (contextual factors, psychosocial factors, and technological factors) at multiple levels (structural, community, household, individual, and habitual) [20]. Semi-structured interviews conducted during formative research were completed in three phases: (1) exploratory research; (2) pilot phase I; and (3) pilot phase II (Figure 1). A total of 93 semi-structured interviews (35 males and 58 females) (50 during the exploratory phase, 43 during phases I and II of the pilot study) were conducted with healthcare providers, diarrhea patients, and mothers, fathers, and other caregivers of young children (e.g., siblings) (Table 1). Research officers received a 1-month qualitative data collection training before conducting semi-structured interviews. All interviews were conducted in Bukavu Swahili. Comprehensive debriefs were completed in French. Transcriptions were completed for all interviews and translated into French and reviewed by at least two research staff to assess accuracy. French transcriptions, summaries, and debrief notes were hand-coded using a codebook that was developed based on exploratory research findings, which was further refined during pilot phases I and II. The codebook was refined based on emerging themes organized using IBM-WASH.

### 2.2. Component 1 Exploratory Research

#### Semi-Structured Interviews

Between February 2020 and November 2020, 50 exploratory interviews were conducted among a purposive sample of 42 diarrhea patients and household members and 8 healthcare workers recruited from health facilities treating severe diarrhea patients in Bukavu. Eligibility requirements for diarrhea patients were: (1) present to a health facility in Bukavu with ≥3 loose stools in a 24-h period; and (2) have no running water (functional tap) in their home. Household members of diarrhea patients had to share the same cooking pot with the diarrhea patient for the past 3 days (definition for a household contact used in previous studies [8,16]). Healthcare providers were included if they worked in a health facility that cared for cholera or severe diarrhea patients. The semi-structured interview guides were designed to explore the IBM-WASH dimensions and levels influencing handwashing with soap, water treatment, and safe water storage behaviors. Questions explored topics such as: cholera and diarrheal disease awareness, and handwashing with soap, water treatment, and safe water storage behaviors in households and health facilities. 

### 2.3. Component 2 Intervention Development and Piloting

#### Starting Point for PICHA7

The starting point for the PICHA7 program followed the model of CHoBI7; however, intervention content and implementation were tailored based on findings from exploratory interviews and the pilot study. The PICHA7 program was designed to target five key behaviors: (1) preparing soapy water for handwashing; (2) handwashing with soap at food- and stool-related events; (3) treating drinking water with chlorine tablets during the 7-day high-risk period; (4) boiling drinking water after the 7-day high-risk period; and (5) storing drinking water safely in a vessel with a lid and tap.

**Figure 2:**
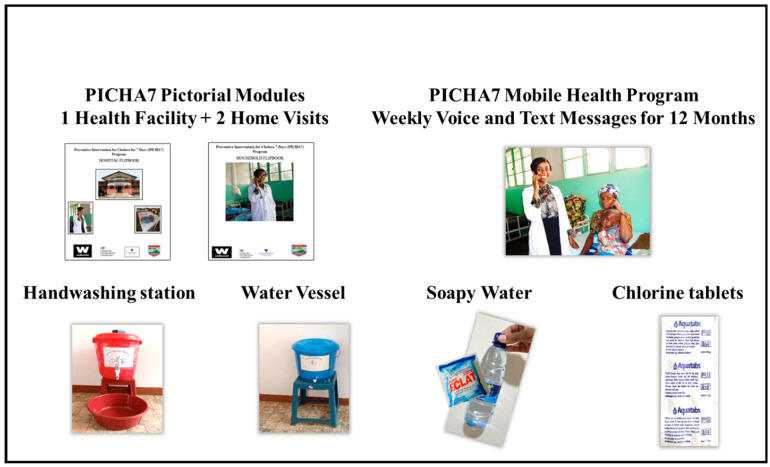
PICHA7 program intervention components and cholera prevention package materials.

The PICHA7 program includes 3 components: (1) pictorial modules (flipbooks) delivered by a health promoter during 1 health facility and 2 household visits (new modules were created for PICHA7); (2) a cholera prevention package, including a handwashing station, soapy water, drinking water vessel with lid and tap, and one month supply of chlorine tablets (WASH enabling technology); (3) 4 cue-to-action cards for target behaviors; and (4) an mHealth program, including voice and text messages delivered weekly for 12 months (3 months for the pilot) (Figure 2). All intervention materials are locally sourced. The health facility pictorial module targets WASH behavior change in both a health facility and a household setting, while the household pictorial module targets WASH behavior change in the household.

### 2.4. Intervention Development

PICHA7 program intervention development targeted the IBM-WASH habitual, individual, household, community, and structural/societal levels at multiple dimensions to ensure that facilitators and barriers of the target WASH behaviors were thoroughly addressed. Intervention development was initially based on exploratory interviews, and further refinements were made based on pilot study findings. The PICHA7 health facility and household pictorial modules and mobile messages were developed during weekly intervention planning workshops over a 14-month period. The workshops included research officers and study investigators. Each workshop was 2–6 hours in length. During initial intervention planning workshops, the key themes that emerged during the exploratory research were discussed and pictorial module content was then developed to target the facilitators and barriers of the behavioral recommendations identified. The mHealth component of the PICHA7 program was developed during intervention planning workshops after the development of initial pictorial modules. mHealth content was developed to align closely with the pictorial modules to target facilitators and barriers to the promoted WASH behaviors identified during exploratory research, also using the IBM-WASH framework. There were three types of mobile messages: interactive voice response (IVR) quizzes, voice calls, and SMS messages. The EngageSpark platform (https://www.engagespark.com/ (accessed on 4 January 2021)) was used to deliver voice, IVR quizzes, and SMS messages for the PICHA7 mHealth program. An mHealth expert from EngageSpark participated in one intervention planning workshop to train the team on how to send mobile messages using this platform. 

### 2.5. Component 3 Pilot Study

Between October 2020 and October 2021, we conducted a two-phase pilot study to explore the feasibility and acceptability of the PICHA7 program. Diarrhea patients (individuals admitted to a health facility with three or more loose stools over a 24-h period) were recruited from the Provincial Hospital of Bukavu. To be eligible for the pilot, diarrhea patient households had to: (1) have at least one household member that shared the same cooking pot with the diarrhea patient for the past 3 days (definition for a household contact used in previous studies [8,16]); (2) have no piped connection for running water inside their home (mostly slum areas of Bukavu); (3) report ownership of an active mobile phone in their possession on the day of enrollment; and (4) have a child under five years of age in their household (to assess the impact of the intervention on pediatric diarrheal disease prevalence). 

**Phase I.** Phase I of the pilot study focused on pre-testing the PICHA7 pictorial modules, mobile messages, and cholera prevention package to determine the initial acceptability and the feasibility of delivering these components over a 1-month period. From October to December 2020, 88 participants from 20 households participated in Phase I piloting. All of the households received the PICHA7 program. Health promoters received 1-month of hands-on training on how to deliver the intervention during the pilot study. Trained health promoters delivered the PICHA7 program during 1 health facility visit during the time of illness for the index diarrhea patient in the household. Household visits were then conducted on Day 2 and Day 5 after enrollment of the patient household. One month after program delivery, semi-structured interviews were conducted to obtain beneficiary feedback on the intervention. The findings from Phase I enabled us to make refinements and adaptations to the PICHA7 program, guided by IBM-WASH, in preparation for a larger and longer 3-month evaluation of the intervention in Phase II. Semi-structured interviews were conducted with a purposive sample of 20 diarrhea patient household members.

**Phase II.** Phase II was a randomized pilot conducted to assess acceptability and feasibility of the refined PICHA7 program over a 3-month period. Four hundred and thirty participants from 85 households participated in Phase II from February to October 2021. Sixty-nine households received the PICHA7 program, with sixteen households enrolled in the standard arm that only received information on the use of oral rehydration solution (ORS) for dehydration. Enrolled households were randomized to the PICHA7 program or standard arm using a block randomized schedule generated by the study biostatistician. Similar to Phase I, households received a health facility visit and two home visits and weekly mobile messages using the same timing for delivery of each component. Five-hour structured observations of handwashing practices at stool- and food-related events in study households were conducted by trained research officers at Day 7, Month 1, and Month 3 after the diarrhea patient was admitted. Additional details on the structured observations methodology are described in our quantitative pilot publication [21]. At the end of the three-month follow-up, intervention household members were interviewed about their experiences with the PICHA7 pictorial modules and cue cards, cholera prevention package, and the mHealth program. Refinements and adaptations to the PICHA7 program were made based on findings from the interviews and IBM-WASH. Semi-structured interviews were conducted with a purposive sample of 23 diarrhea patient household members.

### 2.6. Ethical Approval

Informed written consent was given by all participants for study activities. If the participant was between the ages 12–17 years, the participant provided informed assent and parental or guardian consent was also obtained. For the quantitative pilot study, if the child was 12 years or younger, informed consent was obtained from a parent or guardian. If the participant could not read or write, oral consent was obtained, and a third-party witness was present. This study received ethical approval from the Johns Hopkins Bloomberg School of Public Health and the Catholic University of Bukavu.

## 3. Results

The results presented here are those most salient to the development and refinement of the PICHA7 program. The exploratory research provided insights into experiences with and awareness of cholera, handwashing and water treatment and storage and other hygiene practices, and barriers and facilitators to these WASH behaviors. The pilot study then provided insights on the preferences, acceptability, and feasibility of delivering the PICHA7 program for diarrhea patient households in this setting. 

### 3.1. Exploratory Research

#### 3.1.1. Experiences with Cholera and Cholera Awareness

Many diarrhea patients and household members said they had never heard of cholera before.

*No, before I fell ill, I had never heard of cholera*

*—Male diarrhea patient*

However, for diarrhea patients and household members who had heard of cholera, it was seen as a serious, contagious disease, with participants using words like “brutal”, “dangerous”, and “kills” to describe it. Many said they were scared of how quickly the disease could kill someone, leading to isolation of households with a cholera case out of fear that other community members would become ill.

*On Sunday, I came here with my six-year-old child and yesterday I came with my fifteen-year-old. They both suffer from cholera. People in my community are starting to run away from my house. They say, “We cannot go there, so we do not catch cholera.”*

*—Mother of a patient*

Knowledge of cholera transmission was low and varied widely amongst diarrhea patient household members. Some had heard of cholera but did not know how it was transmitted, while others could only name one or two transmission routes. For those that knew how cholera was transmitted, common responses were by flies, dirt, changes in weather, contaminated water and food, and poor hygiene. Many diarrhea patient household members believed that cholera was spread through bad air or bad spirits rather than water or hygiene.

*From what I’ve heard and what I know, cholera is bad air. It’s not a hygiene problem—because if it was a lack of hygiene, a lot of people could get sick from cholera, because a lot of people have hygiene problems. Cholera is not a problem of lack of hygiene.*

*—Father of young child*

Often participants viewed cholera treatment centers (CTC) and health facilities as high-risk environments for cholera transmission because they were seen as “dirty place(s) with a lot of germs”. One female diarrhea patient explained her family’s fear of visiting her in the CTC:

*My children and colleagues from church come here to visit me. But, because it is at the hospital, they are afraid. It’s [because of this] that they observe me from afar and return home. They think that when they come here to visit someone, they will also become contaminated.*

*—Female patient*

Many diarrhea patients did not know anyone in their community with cholera and believed that cholera was low in their community.

*Currently, I have not yet heard that there is a person in the neighborhood who is suffering from cholera. They only say on the radio that in such and such a neighborhood, people are suffering from cholera, but in our neighborhood, I have not heard yet.*

*—Female household member*

#### 3.1.2. Handwashing Practices

While diarrhea patients and household members said that they knew it was important to wash hands with soap, they also said that people did not because of a strong belief that “Congolese do not die from germs”. 

Often, diarrhea patients and household members found it difficult to remember to wash their hands with soap.

*Sometimes someone can forget because sometimes I get very hungry and when I get home I start to eat before washing my hands. I might remember after eating something without first washing my hands.*

*—Father of young child*

It was also difficult for diarrhea patients and household members to remind their children to wash their hands with soap, and to keep hands clean when at work, in the fields or outside of the home.

*I wash my hands in the morning before eating, but I often eat without washing my hands, especially during the day when I am at the market.*

*—Mother of young child*

Many diarrhea patient household members reported running out of soap for handwashing due to financial insecurity, and some faced challenges finding water for handwashing. While ash was mentioned as an alternative to soap, some mentioned they were hesitant to use this method because they had never tried it before.

*I only use water. If I have soap, I can use it—because there are times when we run out of soap. And when we run out, I don’t have the courage to use ashes to wash my hands.*

*—Father of young child*

Many participants said that they can tell when their hands were clean based on their appearance and the things they had touched. Cleanliness of hands was based on dirt rather than germs.

*I can know for myself that my hands are clean depending on the shopping I’ve done and the things I’ve touched. When I drive all day, I wash my hands because there is a lot of dirt behind the wheel.*

*—Father of young child*

#### 3.1.3. Water Treatment Practices

Many diarrhea patients and household members did not have access to running water near their homes. Many had to travel over 30 min round-trip to collect water for household activities and consumption, with some reporting that it could take up to three hours to collect water. In addition, there were financial constraints on the type of water sources households could use. Because piped water had a cost, some chose untreated spring water instead. 

Even for those paying for piped water, sometimes water was not available from these sources. Most diarrhea patients and household members said that if their water looked clean with no dirt, then it was safe to drink. It was also mentioned that water that came out of the public tap was already treated and therefore safe to drink without treatment.

*There, where we draw water, they often say that the water is already treated. This allows us to use this water without any further treatment.*

*—Father of a patient*

Most diarrhea patients and household members reported that they did not boil or treat their drinking water with chlorine tablets, and that most individuals in their community also did not treat their drinking water. Some had previously boiled drinking water; however, they stopped so that their children could build a tolerance against untreated water.

*I stopped boiling water. The reason for stopping is that when I used to boil water, it was good. But when the children went to the neighbors’ or family members’ [homes] they drank un-boiled water, and when they came back they had abdominal problems. That’s how I left [stopped] boiling water, so that their bellies could get used to un-boiled water.*

*—Mother of a patient*

The aesthetic qualities of chlorinated and boiled water were an important determinant of use of these treatment options. Some diarrhea patient household members stated that chlorinated water had a taste like “medicine”, and that boiled water did not taste as good as untreated water. Despite these findings, most participants agreed that treating water killed microbes. Participants reported that finding and affording chlorine tablets was difficult.

*Even the chlorine, I have never used it. I don’t have it. I don’t have the money to get it and I don’t even know where I can find chlorine.*

*—Father of a young child*

#### 3.1.4. Water Storage Practices

Many diarrhea patient household members reported storing their drinking water in a separate 5L jerry can. Some said they stored drinking water in the bedroom on a high shelf for safe keeping away from children.

*It’s only in this canister. There’s a small 5L canister, and it’s in this canister that I keep the water to drink. That’s where I put the water to drink from. When I go to get water, I also draw from this 5L canister and I keep it inside the house and we start to drink water from that canister.*

*—Diarrhea patient*

Others reported not storing their drinking water separately because the house was too small and there was not enough space. Jerry cans were frequently left uncovered because children often lost the lids when they went to fetch water.

#### 3.1.5. Household Hygiene Practices When a Child Has Diarrhea 

Among diarrhea patient household members, we found that having multiple children sharing the same bed for sleeping was a common practice, even if a child was sick with diarrhea. Some reported that sheets were not washed immediately after becoming soiled with diarrhea by children.

*When I came back [from work] the next morning… I thought to myself, “It looks like my child is having diarrhea”. That’s when his mother informed me that the child had had diarrhea and vomiting during the night, but he slept in the same place with his brothers. I didn’t take the news very seriously, so I went back to work for the day. On my way back, around 12 o’clock, the neighbors informed me that [the child’s] situation had worsened and that he was brought here [the hospital]. So, the neighbors told me to take off the sheets in the house that he had defecated and vomited on and put them in another sack. As their mother had already gone out, I took the sheets off and put them outside in a sack. But they all slept in the same place.*

*—Father of a patient*

#### 3.1.6. WASH in Health Facilities 

There was limited access to safe drinking water in health facilities.

*We have a big problem with drinking water in this hospital because our water treatment companies do not provide us with water that is very clean. The water that flows in our taps is not safe water. And in the hospital, we too do not really have any devices to treat water for it to become safe in order to give to the patients and the people with them, and even for ourselves. It would be really desirable if we had devices to purify the water in order to help everyone be able to drink safe water.*

*—Female doctor*

Some healthcare workers said they provide drinking water treated with chlorine tablets for diarrhea patients. At the local CTC, ORS was also prepared by health workers using boiled water for severe diarrhea patients. 

Water shortages in the health facilities were said to make washing hands difficult. Chlorinated water for handwashing was provided at some handwashing stations in the Provincial General Reference Hospital of Bukavu. However, diarrhea patients and household members were often not aware that the handwashing stations had chlorine and that soap was therefore not needed to clean hands.

*This place reserved for hand washing [where chlorinated water was available] lacks soap. That’s cleanliness and it’s about protecting people from disease.*

*—Female diarrhea patient*

Some diarrhea patients and household members also reported bringing their own soap into health facilities because handwashing stations did not always have soap available. 

### 3.2. Intervention Development and Piloting

In this section, we describe the iterative approach taken in this study for intervention development based on exploratory interviews and pilot study findings. Table 2 shows the framework used for intervention development.

#### 3.2.1. Pictorial Module: Cholera and Severe Diarrheal Disease Transmission 

##### Intervention Development

To address the low awareness of cholera transmission found during exploratory interviews, a cholera and severe diarrhea transmission pictorial module was created for both health facility and household visits, where health promoters explain to diarrhea patients and household members that diarrheal diseases are spread from germs found on unclean hands and in untreated drinking water. The module includes a true story of a family from one of our exploratory interviews, where no one in the household washed hands with soap or drank treated water, and one child died soon after arriving at the CTC with cholera, and a second child became severely ill during the 7-day high-risk period. Because some participants mentioned cholera was caused by dirt rather than germs in exploratory interviews, we placed further emphasis in the pictorial module and the mobile messages explaining that even water that looks clear and hands that have no visible dirt could still have germs that can make household members sick with cholera and severe diarrhea. To address the finding that cholera was not thought to be high in many of the areas where cholera patients were coming from, health promoters explained that cholera was high in their area through a story of a family that came to the pilot participants’ local health facility with cholera.

##### Pilot Findings and Adaptions 

In phase I of the pilot, participants mentioned the value of the story discussed in the pictorial module, and how this helped them to understand the 7-day high-risk period. Knowledge of cholera and severe diarrhea transmission and prevention was much higher among those in the pilot compared to diarrhea patient household members in exploratory interviews.

*Yes, we talked about the 7 days of high risk, we said, when severe diarrhea enters the house or reaches a child, there is another danger that comes after those 7 days. Another child can become ill or others in the household because of this disease and we were shown how this disease spreads and where it can continue to live and when we need to wash our hands.*

*—Father of young child*

#### 3.2.2. Pictorial Module: Handwashing with Soap

##### Intervention Development

A handwashing with soap module that explained the recommended key times for handwashing with soap (before food- and after stool-related events) both in the household and in a health facility setting was created for diarrhea patient households. Because dirt reactivity (i.e., washing hands only when visibly dirty) came up often in exploratory interviews, this module shows that diarrheal disease-causing microbes on hands are invisible to the naked eye, and can be present even if hands look clean. To address concerns among participants about the affordability of bar soap, during the health facility and home visits, research officers demonstrated to households how to make soapy water from water and detergent powder (1/10th the cost of bar soap), with household members preparing soapy water themselves during this demonstration. Health promoters also mentioned at the final household visit that ash is a free substitute for soap that can be used for handwashing, and had an ash demonstration. To encourage children in the household to wash hands with soap, we developed a song sung in the melody of a local nursery rhyme about when and how to wash hands with soap or ash. Lastly, to address the finding that remembering the key times for handwashing was difficult, a cue-to-action card showing the key times to wash hands with soap was created to place on the wall near the household’s handwashing station to serve as a reminder to perform these behaviors. 

##### Pilot Findings and Adaptions 

Participants reported having difficulty washing their hands with soap before eating when they were outside the home.

*For people who buy things along the way, it could be a mango or a donut, someone could buy it and eat it without remembering to wash their hands… It depends on the environment, but also the conditions in which we live, because there are those who are not used to washing their hands.*

*—Mother of a young child*

To address this, we recommended bringing the small soapy water bottle we provided when participants leave their home, and to use the many handwashing stations already present in Bukavu because of the ongoing COVID-19 pandemic. There was also confusion as to whether handwashing with soap was needed when eating small snacks, such as donuts and fruit. Following this, we reinforced the need to wash hands with soap before eating any food, even snacks.

In the beginning of phase II, handwashing with soap at key events in diarrhea patient households was low on Day 7 (32%) during 5-h structured observations, which was consistent with interview findings, where participants said that they were no longer at risk for cholera because it was the end of the 7-day high-risk period.

*I had heard you say that if a child suffers from cholera on the first day, the second day until the seventh day [after they come to the health facility], if there is not anyone with cholera after those seven days... no one can catch [cholera] anymore.*

*—Mother of a young child*

To reiterate the importance of handwashing with soap as a lifelong practice, we moved the second household intervention visit from the fifth day after health facility admission to the seventh day to align with the end of the 7-day high-risk period for cholera. During this final visit, households were congratulated for performing the promoted WASH behaviors during the 7-day high-risk period and health promoters explained that these should be lifelong practices. We also added the true story of one enrolled family who stopped handwashing with soap and water treatment after the 7-day high-risk period, and sadly had to return to the CTC after their second child fell ill with severe diarrhea a few weeks later. In addition, to provide more detail and photos on the key events for handwashing with soap, this module was divided into two sections: (1) handwashing with soap at food-related events; and (2) handwashing with soap at stool-related events. After implementing these changes, by the end of phase II, we observed that 58% of household members were washing their hands at least once during structured observations at Day 7.

#### 3.2.3. Pictorial Module: Water Treatment and Storage

##### Intervention Development

The pictorial module on water treatment and storage recommended treating drinking water with chlorine tablets during the 7-day high-risk period and to chlorinate *or* boil drinking water thereafter. To address the difficulty that participants reported with finding chlorine tablets locally, we added a section to the water treatment and storage module showing photos of locally available chlorine tablets, and named the locations and showed photos of where they could be purchased. To address the concerns around the taste of treated water, we explained that the taste of boiled and chlorine-treated water indicated that the water had been treated and was now safe to drink. We also explained that just as bitter gourd (a local vegetable) had a bitter taste but was very healthy, chlorine-treated and boiled water may have a taste but that this water was important for preventing diseases and keeping one’s family healthy. To address affordability concerns about the chlorine tablets, we noted that chlorine tablets were 1/8 the cost of boiling water.

##### Pilot Findings and Adaptions 

Some pilot participants believed that if they drank chlorine-treated water this could act as a medicine and would protect them from becoming sick, even if they ate food contaminated with germs.

*They will be able to know the change through this medicine. It is a good medicine. For us who have children in this environment, the child can pick up a doughnut beignet outside, but if he has drunk this water, we will be quiet because we know he has taken the medicine. This medicine will help. The microbe can enter and be eliminated*

*—Female household member*

Following this, we modified the pictorial flipbook to highlight that chlorine-treated water is not a medicine and only protects the drinking water where the tablet is used and cannot protect household members from diarrheal diseases transmitted via other routes. 

In phase II, we identified confusion on how to boil water and when to use it—some pilot participants thought the temperature indicated whether water was boiled properly, and others thought water treated by boiling or chlorine tablets should be used in both the safe drinking water container and the handwashing station. To address these findings, we further emphasized in the pictorial modules and mobile messages that water must be boiled until large bubbles formed, and that untreated water could be used in the handwashing station. Although pilot participants were able to find chlorine tablets locally, some said that locally available ones were of inferior quality compared to ones provided during the intervention. To address this, we measured the chlorine concentrations in two commonly avaliable chlorine tablets in the local market and found that they were of equal quality to the ones provided in the intervention (aquatabs). We showed photos of this comparison and conveyed the equivalence to participants during intervention delivery. 

#### 3.2.4. Household Hygiene Practices when a Child has Diarrhea 

##### Intervention Development

Based on the finding during the exploratory research that it was common for children with diarrhea and healthy children to sleep together, we created a module about how this could potentially spread diarrheal diseases. The module tells the true story of two siblings who slept in the same bed as their brother, who was sick with severe diarrhea. The next day, all three young children were admitted to the CTC. The target behaviors for this module are: (1) having children sick with diarrhea sleep separately from healthy children (with the caregiver, in an extra bed, or on a sheet next to the bed) or for the caregiver to use a diaper for this child, and (2) washing clothing, diapers, and sheets that are soiled with feces with soap or detergent immediately after they are soiled, and cleaning hands thoroughly after with soap, soapy water, or ash. Pilot participants did not mention barriers to performing this behavior during piloting.

#### 3.2.5. PICHA7 mHealth Program

##### Intervention Development

Twelve IVR quizzes and corresponding summary SMS messages, and twelve voice calls with corresponding summary SMS messages, were developed aligning with the pictorial modules and behavioral recommendations delivered during health facility and household visits (Supplementary File S1). Following the CHoBI7 model [13], two characters were created to deliver the PICHA7 mHealth program: “Dr. Picha”, a doctor at a hospital who treats cholera and severe diarrhea patients, and “Mwanza”, a mother of a young child that fell ill with cholera and needed to go a hospital for treatment. Messages were sent in the late afternoon between 4 and 6 PM in pilot phase I, so that they could be delivered when household members returned home from work. 

##### Pilot Phase Findings and Adaptions 

We found that pilot participants preferred to receive mHealth messages in the evenings rather than in the late afternoon.

*I prefer the time when my parents are already home from work, when we are all together in the same place, maybe in the evening, because you can send them (messages) during the day when he [father] is still at work, and in the evening when he arrives home, he forgets to share them with us.*

*—Male household member*

Based on this feedback, we changed the timing of mHealth messages to later in the evening, between 7pm and 9pm. 

Pilot participants also reported difficulty responding to IVR quiz messages. In response to this finding, health promoters spent more time during health facility and home visits explaining how to respond to IVR quiz messages and played recorded messages on the health promoter’s phone. Pilot participants also reported difficulty understanding the language of some voice and SMS messages.

*There were not any messages we didn’t like, but there were some words that we didn’t understand, maybe [some] terms we didn’t understand*

*—Female diarrhea patient*

As a result, we reviewed mobile message content with participants and made revisions for clarity, using more easily understood terms for those with no or a few years of formal education. We also spoke more slowly in recordings and added additional pauses. 

In phase II, participants continued to express difficulty with IVR quizzes. To address this, the PICHA7 intervention team expanded the mHealth training session for participants further by sending IVR quiz messages directly to participants’ phones during each in-person visit; this gave participants hands-on training on how to respond to IVR quizzes. Pilot participants said that mobile messages served as reminders of the promoted WASH behaviors.

*I liked these [mobile] messages because they remind us if we have already forgotten.*

*—Father of a patient*

#### 3.2.6. Cholera Prevention Package

##### Intervention Development

As indicated in Figure 2, the cholera prevention package includes a handwashing station, soapy water, drinking water vessel with lid and tap, and one month supply of chlorine tablets.

##### Pilot Findings and Adaptions

Most pilot participants preferred the PICHA7 safe drinking water vessel over the typically used 20L jerry cans because the lids of jerry cans were often lost by children, whereas the lid on the safe drinking water storage vessel was sturdy and did not let dirt in. 

Some pilot participants reported difficulty finding the same detergent powder in the market that we had provided for soapy water during health facility and household visits—they were concerned that if they bought a different detergent powder, the soapy water solution would not be the same quality. Participants also said that the detergent powder they found in the market did not have the same smell. Based on this finding, we emphasized in flipbooks that soapy water could be made with any type of detergent powder. We also provided additional detergent powder in an unmarked bag so that participants would not think that they needed to purchase the same brand we provided. Soap or soapy water being present next to the handwashing station emerged as an important reminder to wash hands with soap.

*It is difficult to wash hands with soap when the soap is not next to the handwashing station. When it is not there, not everyone will remember it. But when the person sees that soap is near the handwashing station at all times, it will allow him or her to wash with soap.*

*—Male diarrhea patient*

Intermittent water access continued to be a challenge in the pilot study, with pilot participants reporting that their buckets were running out of water.

*It may seem to be enough but the amount of water doesn’t last. The water inside this bucket doesn’t last.*

*—Household member*

To address this, a jerry can was provided to allow for additional water storage, and a tap with a slower flow rate was attached to the handwashing station to reduce the amount of water required for handwashing.

We observed high acceptability of the cholera prevention package.

*These materials help me a lot. When the children want to wash their hands they go there (PICHA7 hand washing station). If they want to drink water, they take from this blue bucket (PICHA7 drinking water vessel).*

*—Mother of young child*

Some participants asked for an extra handwashing station or drinking water station to give to their neighbors. We encouraged these participants to show their neighbors how to prepare these materials themselves. Demonstrations by health promoters were also performed during health facility and household visits on how to make a handwashing station and a drinking water vessel (making a hole in a bucket and inserting a tap); for demonstrations, household members performed these activities under the guidance of the health promoter to allow them to receive hands-on training.

## 4. Discussion

Formative research was conducted to inform the design and content of the PICHA7 program to reduce cholera and severe diarrhea transmission among household members of diarrhea patients in the DRC. Awareness of cholera and severe diarrhea transmission and prevention was low in the community. This led to the inclusion of a true story in a pictorial module of a family where one child died soon after arriving to a CTC with cholera, and a second child became severely ill during the 7-day high-risk period (after the initial child became ill). The pictorial module explained the potential transmission pathways by which household members could become sick with cholera and severe diarrhea. Children sharing the same bed with a child with diarrhea emerged as a common practice and a potential transmission route for cholera and severe diarrheal disease, which was targeted as part of the PICHA7 program through another true story of a family that came to the CTC. Soapy water emerged as a promising low-cost alternative to bar soap in our study setting. PICHA7 mHealth messages served as important reminders to perform the promoted WASH behaviors, however the timing of mobile message delivery had to be tailored to the schedule of households, and extensive training was needed on how to respond to IVR quiz messages. Modifications were also needed to the cholera prevention package to provide additional water storage, and to reduce the amount of water needed for handwashing. Overall, the PICHA7 program had high acceptability and was feasible to implement in our study setting. These findings complement our quantitative randomized pilot, which found that PICHA7 significantly reduced diarrhea and led to sustained increases in handwashing with soap and water treatment practices during the 3-month pilot period [2]. This formative research provided important insights into the health facility and household environment, which allowed for the PICHA7 program to be tailored to our setting in the DRC.

Despite the global burden of cholera, particularly in sub-Saharan Africa, and the high risk for cholera transmission for the household members of cholera patients, there was limited awareness of cholera transmission and prevention in our study setting in DRC [2,7,8]. This is consistent with previous findings from our research group and other studies conducted in Bangladesh [22,23,24,25]. One study in Zanzibar found that rural women were less likely to be able to specify cholera transmission routes than male counterparts [26]. Two studies in the DRC and Zanzibar found low cholera awareness despite ongoing cholera outbreaks [26,27]. Similar to our study, in Nigeria, many mothers thought diarrhea was caused by spirits [28]. In our REDUCE cohort study in rural eastern DRC, we found that caregiver diarrheal disease awareness was associated with caregiver handwashing with soap assessed by structured observation [29]. In addition, in our RCT of the CHoBI7 program for cholera patient households in Bangladesh, cholera awareness was a significant mediator of sustained handwashing with soap (assessed by structured observation) at the 6- to 12-month follow-up [30]. These findings demonstrate the need to target diarrheal disease awareness as part of evidence-based theory-informed approaches for delivering WASH programs to facilitate WASH behavior change.

In our study setting, young children with diarrhea were frequently defecating in beds shared with other young children, presenting a potential exposure pathway to fecal pathogens. Previous studies have identified close contact and bed sharing with patients as a transmission route for infectious diseases but have not explicitly focused on bed sharing for those with cholera and severe diarrheal diseases [31,32]. In the PICHA7 program, we encouraged caregivers of young children to not have children with diarrhea sleep in the same bed as healthy children in the household or for the sick child to wear a diaper. We also emphasized the need for clothing and linen for those with diarrhea in the household to be cleaned immediately after being soiled with diarrhea. Future studies are needed to investigate this potential transmission pathway for diarrheal diseases, and possible interventions.

Narratives have been a valuable tool for health promotion in public health through motivating behavior change [33,34,35,36,37,38,39]. This approach has frequently been used for smoking cessation programs [33,34,36], sexual health promotion [40,41], and cancer prevention and control [42,43]. The PICHA7 program used firsthand experiential stories told during participant interviews to explain transmission routes and preventative measures for cholera and severe diarrheal diseases. These narratives made the program content more personal to participants by describing the lived experiences of other diarrhea patient households like themselves. The use of narratives has also been adapted to promote health behaviors through game-based interventions [38]. A recent meta-analysis found that game-based interventions that included narratives were more effective in changing knowledge, self-efficacy, and health behaviors. WASH interventions, however, typically do not use this method for behavior change. More research is needed to determine the effectiveness of narratives implemented as part of WASH interventions. 

Implementing a theory-driven approach for intervention development, informed by IBM-WASH, allowed us to organize facilitators and barriers to WASH behavior change by type of factor (contextual, psychosocial, and technological), and by level (community, household, individual, habitual). This framework provided valuable guidance for intervention development through allowing us to develop targeted interventions at multiple levels. Descriptive norms, dirt reactivity, knowledge, and remembering were all psychosocial factors found to be influential in driving WASH behaviors in our setting. We also identified technological factors, such as the availability of soap and water for handwashing, and access to chlorine tablets. Through identifying these factors, we were then able to tailor our WASH behavior change communication program to target them. If we had provided the same CHoBI7 program delivered in Bangladesh in the DRC, without conducting formative research, we would have missed important context-specific technological and psychosocial factors, including water scarcity, beliefs around cholera transmission, and perceived susceptibility to cholera and severe diarrhea. Our approach to intervention development is similar to our previous work for the development of the CHoBI7 program in Bangladesh, and our REDUCE program in the DRC, which promoted handwashing with soap, water treatment, playmats to reduce child mouthing of dirt, and safe disposal of child and animal feces [12,13,44]. A similar formative study was also conducted in Bangladesh to design the handwashing station we used in our current study [45]. Human-centered design for WASH programs has been shown to help identify barriers and facilitators to consistent and sustained use of WASH enabling technologies [46,47,48]. Future studies are needed that use IBM-WASH to tailor programs to the community context in other settings globally to facilitate WASH behavior change.

This study has several strengths. First, a key strength of PICHA7 program development is the theory-informed and evidence-based approach for program design, which tailored intervention content and delivery to the identified barriers and facilitators of the promoted WASH behaviors. We used the IBM-WASH framework to consider multiple levels and dimensions of WASH behavior change, not just the individual level. Second, the PICHA7 program includes the use of narratives, allowing the beneficiary to relate to the intervention content through the lived experiences of people like themselves. Third, having two pilot phases allowed for an iterative process of intervention development based on beneficiary feedback. Fourth, the mHealth component is a novel approach to promote behavior change in the WASH field and in this setting. 

This study has some limitations. First, we did not conduct focus group discussions due to physical distancing requirements during the COVID-19 pandemic. Focus group discussions could have allowed for a more participatory approach for developing and finalizing intervention materials. Second, we only assessed acceptability and feasibility of the intervention over a 3-month period. Future studies should follow participants for longer periods of time to investigate facilitators and barriers of sustained WASH behavior change. Third, we conducted this formative research in an urban setting in Eastern DRC. The PICHA7 program could also be adapted and delivered in rural areas of South Kivu, DRC, and in hotspots elsewhere in DRC; however, additional formative research will be needed to do this. 

## 5. Conclusions

WASH interventions that are tailored to target specific populations and contexts are needed to facilitate WASH behavior change. The PICHA7 program used formative research to develop a targeted WASH intervention to reduce cholera and severe diarrhea among patient households in urban Eastern DRC. The study identified several context-specific factors, including intermittent access to water which limited water for handwashing and for water treatment using chlorine tablets, unaffordability of soap for handwashing, and low awareness of cholera transmission and prevention. These findings aided in adapting the PICHA7 program to our setting. This research can provide a model for the adaption of future WASH programs in different contexts. The PICHA7 program presents a promising approach to increasing WASH behaviors among diarrhea patient households in urban eastern DRC.

## Figures and Tables

**Table 1:** Demographic characteristics of interviewees.

	Exploratory	Pilot Phase 1	Pilot Phase 2
*Total interviewed*	50	20	23
*Participant type*			
*Diarrhea patient*	8	3	0
*Household member*	33	17	23
*Health provider*	9	0	0
*Mean Age (Years)*	33	34	30
*Female*	30	11	17

**Table 2:** Technological, contexual, and psychosocial factors targeted in the Development of the Preventative-Intervention-for-Cholera-for-7-Days (PICHA7) program.

Factor Type(s) Targeted	Description of Factor	Level	Implications for Intervention Design
***Cholera Transmission***			
Psychosocial *Beliefs* *Knowledge*	Belief that cholera is spread through bad air or spirits	Individual	A health promoter delivers a pictorial module and shares the true story of a family with a child that died of cholera to explain that cholera is spread from germs found on unclean hands and in untreated drinking water.
Psychosocial *Knowledge* *Perceived Susceptibility*	Cholera and severe diarrheal diseases are thought to be low in the community	Individual	A health promoter explains that cholera is high in the participants’ neighborhood/area and that another nearby family recently came to the local health facility with cholera.
***Treating Drinking Water***			
Psychosocial *Perceived Susceptibility*	Belief that tap water has already been treated and is therefore safe to drink	Individual	A health promoter delivers a pictorial module and mobile messages are sent explaining that even if water is treated at the plant, often the pipes have holes because the water system is old in Bukavu, which can contaminate the water and make it unsafe to drink.
Psychosocial *Perceived Susceptibility*	Belief that children that drink untreated water can build a tolerance that prevents them from getting sick	Individual	A health promoter delivers a pictorial module and mobile messages are sent explaining that household members including children can fall ill with cholera and severe diarrhea and that drinking untreated water does not help build a tolerance against severe diarrheal diseases.
Technological *Access, availability, and affordability of product* *Household wealth*	Access to and availability of chlorine tablets can be difficult; cost of chlorine tablets may be prohibitive	Community Household	A health promoter delivers a pictorial module and mobile messages are sent explaining that chlorine products are available locally and the locations where they can be purchased. A health promoter delivers a pictorial module and mobile messages are sent that explain how to properly boil water if households cannot find or afford chlorine tablets. Provision of a one-month’s supply of chlorine tablets.
Psychosocial *Dirty reactivity*	If water looks clear, then it is clean and safe to drink	Individual	A health promoter delivers a pictorial module and mobile messages explaining that water that looks clear can still have germs that cause diarrheal diseases and that germs are not visible to the eyes.
Psychosocial *Descriptive Norms*	Perception that no one boils water in the neighborhood	Individual	A health promoter explains that, since starting the PICHA7 program, many households are now treating their drinking water in their neighborhood.
Technological *Perceived low quality of available product*	Concern that some commonly available chlorine tablets in the market were of inferior quality	Individual	Chlorine testing was performed of two commonly available chlorine tablets in the market. Both had similar chlorine concentrations to the ones provided in the PICHA7 program. A health promoter showed photos of these chlorine products in the pictorial module and explained that we tested them in the laboratory and they were the same quality as the program-provided ones.
***Handwashing with Soap***			
Contextual & Technological *Access and availability of water* *Household infrastructure*	Intermittent water access	Community Household	A jerry can is provided to allow for additional water storage, and a tap with a slower flow rate is attached to the handwashing station to reduce the amount of water required for handwashing.
Psychosocial *Perceived Susceptibility* *Perceived Severity*	Belief that Congolese do not need to wash their hands with soap because they do not die from germs	Individual	A health promoter shares the true story of a family from the exploratory interviews, where the household does not treat their water and the children fall ill with cholera, and one child dies.
Psychosocial *Remembering*	It is difficult to remember to wash hands with soap, especially for children	Individual	A health promoter teaches household members a handwashing song for children, reinforcing how and when to wash hands, and the benefits of handwashing with soap or ash.
Contextual & Technological *Household wealth* *Perceived cost/value of product*	Soap is expensive and it can be difficult to have enough soap for handwashing	Household Individual	Demonstration by health promoter on how to make soapy water (low-cost alternative to bar soap). Demonstration by health promoter on how to wash hands with ash (no cost alternative to bar soap). Provision of soapy water during the 7-day high-risk period for cholera after the patient in the household falls ill.
Psychosocial *Dirt Reactivity*	Visibly clean hands don’t have germs on them	Individual	A health promoter delivers a pictorial modules and mobile messages are sent explaining that hands that appear clean can still have germs on them that cause diarrheal diseases.
***Household Hygiene Practices When a Child Has Diarrhea***			
Contextual *Available space*	Young children with diarrhea sleep in the same spaces as their siblings	Household	Health promoter tells the true story of a child with diarrhea sharing the same bed as other children, and afterwards the other children became sick with severe diarrhea and had to go to the health facility. A health promoter delivers a pictorial module encouraging households to have children sick with diarrhea sleep separately from healthy children (with the caregiver, in an extra bed, or on a sheet next to the bed) or for the caregiver to use a diaper for this child.

## Data Availability

Anonymized data may be made available upon request. Requests should be directed to Christine Marie George (cmgeorge@jhu.edu).

## Supplementary Materials

The following supporting information can be downloaded at: https://www.mdpi.com/article/10.3390/ijerph191912243/s1, Supplementary File S1.

## References

[1] Ali M., Nelson A.R., Lopez A.L., Sack D.A. (2015). Updated Global Burden of Cholera in Endemic Countries. PLoS Negl. Trop. Dis..

[2] GTFCC (2017). Ending Cholera: A Global Roadmap to 2030.

[3] Muyembe J.J., Bompangue D., Mutombo G., Akilimali L., Mutombo A., Miwanda B., Mpuruta J.D., Deka K.K., Bitakyerwa F., Saidi J.M. (2013). Elimination of cholera in the democratic Republic of the Congo: The new national policy. J. Infect. Dis..

[4] Bompangue D., Giraudoux P., Handschumacher P., Piarroux M., Bertrand S., Ekwanzala M., Kebela I., Piarroux R. (2008). Lakes as a source of cholera in DRC. Emerg. Infect. Dis..

[5] Bompangue D., Giraudoux P., Piarroux M., Mutombo G., Shamavu R., Sudre B., Mutombo A., Mondonge V., Piarroux R. (2009). Cholera epidemics, war and disasters around Goma and Lake Kivu: An eight-year survey. PLoS Negl. Trop. Dis..

[6] Lessler J., Moore S.M., Luquero F.J., McKay H.S., Grais R., Henkens M., Mengel M., Dunoyer J., M’bangombe M., Lee E.C. (2018). Mapping the burden of cholera in sub-Saharan Africa and implications for control: An analysis of data across geographical scales. Lancet.

[7] Azman A.S., Luquero F.J., Salje H., Mbaibardoum N.N., Adalbert N., Ali M., Bertuzzo E., Finger F., Toure B., Massing L.A. (2018). Micro-Hotspots of Risk in Urban Cholera Epidemics. J. Infect. Dis..

[8] Weil A.A., Khan A.I., Chowdhury F., Larocque R.C., Faruque A.S., Ryan E.T., Calderwood S.B., Qadri F., Harris J.B. (2009). Clinical outcomes in household contacts of patients with cholera in Bangladesh. Clin. Infect. Dis..

[9] Clemens J.D., Nair G.B., Ahmed T., Qadri F., Holmgren J. (2017). Cholera. Lancet.

[10] D’Mello-Guyett L., Cumming O., Bonneville S., D’Hondt R., Mashako M., Nakoka B., Gorski A., Verheyen D., Van den Bergh R., Welo P.O. (2021). Effectiveness of hygiene kit distribution to reduce cholera transmission in Kasaï-Oriental, Democratic Republic of Congo, 2018: A prospective cohort study. BMJ Open.

[11] Deb B., Sircar B., Sengupta P., De S., Mondal S., Gupta D., Saha N., Ghosh S., Mitra U., Pal S. (1986). Studies on interventions to prevent eltor cholera transmission in urban slums. Bull. World Health Organ..

[12] Thomas E.D., Zohura F., Hasan M.T., Rana M.S., Teman A., Parvin T., Masud J., Bhuyian M.S.I., Hossain M.K., Hasan M. (2020). Formative research to scale up a handwashing with soap and water treatment intervention for household members of diarrhea patients in health facilities in Dhaka, Bangladesh (CHoBI7 program). BMC Public Health.

[13] George C.M., Zohura F., Teman A., Thomas E., Hasan T., Rana S., Parvin T., Sack D.A., Bhuyian S.I., Labrique A. (2019). Formative research for the design of a scalable water, sanitation, and hygiene mobile health program: CHoBI7 mobile health program. BMC Public Health.

[14] George C.M., Ahmed S., Talukder K.A., Azmi I.J., Perin J., Sack R.B., Sack D.A., Stine O.C., Oldja L., Shahnaij M. (2015). Shigella Infections in Household Contacts of Pediatric Shigellosis Patients in Rural Bangladesh. Emerg. Infect. Dis..

[15] Black R.E., Merson M.H., Rowe B., Taylor P.R., Abdul Alim A.R., Gross R.J., Sack D.A. (1981). Enterotoxigenic Escherichia coli diarrhoea: Acquired immunity and transmission in an endemic area. Bull. World Health Organ..

[16] George C.M., Monira S., Sack D.A., Rashid M.U., Saif-Ur-Rahman K.M., Mahmud T., Rahman Z., Mustafiz M., Bhuyian S.I., Winch P.J. (2016). Randomized Controlled Trial of Hospital-Based Hygiene and Water Treatment Intervention (CHoBI7) to Reduce Cholera. Emerg. Infect. Dis..

[17] Amin N., Pickering A.J., Ram P.K., Unicomb L., Najnin N., Homaira N., Ashraf S., Abedin J., Islam M.S., Luby S.P. (2014). Microbiological evaluation of the efficacy of soapy water to clean hands: A randomized, non-inferiority field trial. Am. J. Trop. Med. Hyg..

[18] George C.M., Jung D.S., Saif-Ur-Rahman K.M., Monira S., Sack D.A., Rashid M.U., Mahmud T., Mustafiz M., Rahman Z., Bhuyian S.I. (2016). Sustained Uptake of a Hospital-Based Handwashing with Soap and Water Treatment Intervention (Cholera-Hospital-Based Intervention for 7 Days [CHoBI7]): A Randomized Controlled Trial. Am. J. Trop. Med. Hyg..

[19] George C.M., Monira S., Zohura F., Thomas E.D., Hasan M.T., Parvin T., Hasan K., Rashid M.U., Papri N., Islam A. (2020). Effects of a Water, Sanitation and Hygiene Mobile Health Program on Diarrhea and Child Growth in Bangladesh: A Cluster-Randomized Controlled Trial of the CHoBI7 Mobile Health Program. Clin. Infect. Dis..

[20] Dreibelbis R., Winch P.J., Leontsini E., Hulland K.R., Ram P.K., Unicomb L., Luby S.P. (2013). The Integrated Behavioural Model for Water, Sanitation, and Hygiene: A systematic review of behavioural models and a framework for designing and evaluating behaviour change interventions in infrastructure-restricted settings. BMC Public Health.

[21] Christine Marie George A.M., Camille Williams J.-C.B., Presence Sanvura K.E., Elizabeth Thomas J.P., Cirhuza Cikomola J.B., Ghislain Maheshe L.B. (2022). Randomized Pilot of the Preventative Intervention for Cholera for 7 Days (PICHA7) Program on Diarrheal Disease and Handwashing with Soap and Water Treatment in the Democratic Republic of the Congo. Submitt. Environ. Health.

[22] Masud J., Islam Bhuyian M.S., Kumar Biswas S., Zohura F., Perin J., Papri N., Dil Farzana F., Parvin T., Monira S., Alam M. (2020). Diarrhoeal disease knowledge among diarrhoea patient housholds: Findings from the randomised controlled trial of the Cholera-Hospital-Based-Intervention-for-7-days (CHoBI7) mobile health program. Trop. Med. Int. Health.

[23] Saif-Ur-Rahman K.M., Alam M., Sack R.B., Parvin T., George C.M., Zohura F., Shaly N.J., Bhuyian S.I., Rashid M.-U., Begum F. (2016). Promotion of Cholera Awareness Among Households of Cholera Patients: A Randomized Controlled Trial of the Cholera-Hospital-Based-Intervention-for-7 Days (CHoBI7) Intervention. Am. J. Trop. Med. Hyg..

[24] Tamason C.C., Tulsiani S.M., Siddique A.K., Hoque B.A., Jensen P.K.M. (2016). What is cholera? A preliminary study on caretakers’ knowledge in Bangladesh. J. Health Popul. Nutr..

[25] Wahed T., Kaukab S.S.T., Saha N.C., Khan I.A., Khanam F., Chowhury F., Saha A., Khan A.I., Siddique A.U., Cravioto A. (2013). Knowledge of, attitudes torward, and preventative practices relating to cholera and oral cholera vaccine among urban high-risk groups: Findings of a cross-sectional study in Dhaka, Bangladesh. BMC Public Health.

[26] Schaetti C., Khatib A.M., Ali S.M., Hutubessy R., Chaignat C.L., Weiss M.G. (2010). Social and cultural features of cholera and shigellosis in peri-urban and rural communities of Zanzibar. BMC Infect. Dis..

[27] Merten S., Schaetti C., Manianga C., Lapika B., Chaignat C.-L., Hutubessy R., Weiss M.G. (2013). Local perceptions of cholera and anticipated vaccine acceptance in Katanga province, Democratic Republic of Congo. BMC Public Health.

[28] Dikassa L., Mock N., Magnani R., Rice J., Abdoh A., Mercer D., Bertrand W. (1993). Maternal behavioural risk factors for severe childhood diarrhoeal disease in Kinshasa, Zaire. Int. J. Epidemiol..

[29] Bisimwa L., Endres K., Williams C., Thomas E.D., Kuhl J., Coglianese N., Bauler S., Masud J., François R., Saxton R. (2022). Diarrheal Disease Awareness Is Associated with Caregiver Handwashing with Soap in the Democratic Republic of the Congo (REDUCE Program). Am. J. Trop. Med. Hyg..

[30] George C.M., Biswas S., Jung D., Perin J., Parvin T., Monira S., Saif-Ur-Rahman K.M., Rashid M.U., Bhuyian S.I., Thomas E.D. (2017). Psychosocial Factors Mediating the Effect of the CHoBI7 Intervention on Handwashing With Soap. Health Educ. Behav..

[31] Islam M.S., Luby S.P., Sultana R., Rimi N.A., Zaman R.U., Uddin M., Nahar N., Rahman M., Hossain M.J., Gurley E.S. (2014). Family caregivers in public tertiary care hospitals in Bangladesh: Risks and opportunities for infection control. Am. J. Infect. Control.

[32] Ngale K.M., Santos I.S., Gonzalez-Chica D.A., de Barros A.J., Matijasevich A. (2013). Bed-sharing and risk of hospitalisation due to pneumonia and diarrhoea in infancy: The 2004 Pelotas Birth Cohort. J. Epidemiol. Community Health.

[33] Cherrington A., Williams J.H., Foster P.P., Coley H.L., Kohler C., Allison J.J., Kiefe C.I., Volkman J.E., Houston T.K. (2015). Narratives to enhance smoking cessation interventions among African-American smokers, the ACCE project. BMC Res. Notes.

[34] Kim H.S., Bigman C.A., Leader A.E., Lerman C., Cappella J.N. (2012). Narrative Health Communication and Behavior Change: The Influence of Exemplars in the News on Intention to Quit Smoking. J. Commun..

[35] Remein C.D., Childs E., Pasco J.C., Trinquart L., Flynn D.B., Wingerter S.L., Bhasin R.M., Demers L.B., Benjamin E.J. (2020). Content and outcomes of narrative medicine programmes: A systematic review of the literature through 2019. BMJ Open.

[36] Fu S.S., Rhodes K.L., Robert C., Widome R., Forster J.L., Joseph A.M. (2014). Designing and evaluating culturally specific smoking cessation interventions for American Indian communities. Nicotine Tob. Res..

[37] Laskow T., Small L., Wu D.S. (2019). Narrative Interventions in the Palliative Care Setting: A Scoping Review. J. Pain Symptom Manag..

[38] Zhou C., Occa A., Kim S., Morgan S. (2020). A Meta-analysis of Narrative Game-based Interventions for Promoting Healthy Behaviors. J. Health Commun..

[39] Hinyard L.J., Kreuter M.W. (2007). Using narrative communication as a tool for health behavior change: A conceptual, theoretical, and empirical overview. Health Educ. Behav..

[40] (1999). Community-level HIV intervention in 5 cities: Final outcome data from the CDC AIDS Community Demonstration Projects. Am. J. Public Health.

[41] Mevissen F.E.F., Ruiter R.A.C., Meertens R.M., Schaalma H.P. (2010). The effects of scenario-based risk information on perceptions of susceptibility to Chlamydia and HIV. Psychol. Health.

[42] Sitto K., Lubinga E., Geya M. (2021). The power of narrative health communication: Exploring possible effects of first-hand experiential stories on cancer awareness amongst university students. J. Transdiscipl. Res. South. Afr..

[43] Kreuter M.W., Green M.C., Cappella J.N., Slater M.D., Wise M.E., Storey D., Clark E.M., O’Keefe D.J., Erwin D.O., Holmes K. (2007). Narrative communication in cancer prevention and control: A framework to guide research and application. Ann. Behav. Med..

[44] Kuhl J., Bisimwa L., Thomas E.D., Williams C., Ntakirutimana J., Coglianese N., Bauler S., François R., Sanvura P., Bisimwa J.C. (2021). Formative research for the development of baby water, sanitation, and hygiene interventions for young children in the Democratic Republic of the Congo (REDUCE program). BMC Public Health.

[45] Hulland K.R., Leontsini E., Dreibelbis R., Unicomb L., Afroz A., Dutta N.C., Nizame F.A., Luby S.P., Ram P.K., Winch P.J. (2013). Designing a handwashing station for infrastructure-restricted communities in Bangladesh using the integrated behavioural model for water, sanitation and hygiene interventions (IBM-WASH). BMC Public Health.

[46] Devine J., Karver J., Coombes Y., Chase C., Hernandez O. (2012). Behavioral Determinants of Handwashing with Soap among Mothers and Caretakers: Emergent Learning from Senegal and Peru.

[47] Devine J., Karver J., Coombes Y., Chase C., Hernandez O. (2012). Global Scaling Up Handwashing Project Behavioral Determinants of Handwashing with Soap Among Mothers and Caretakers: Emergent Learning from Senegal and Peru.

[48] Coombes Y., Devine J. (2010). Global Scaling Up Handwashing Project: Introducing FOAM-a Framework to Analyze Handwashing Behaviors to Design Effective Handwashing Programs.

