# Supplementary material for: Formative Research for the Development of Evidence-Based Targeted Water, Sanitation, and Hygiene Interventions to Reduce Cholera in Hotspots in the Democratic Republic of the Congo: Preventative Intervention for Cholera for 7 Days (PICHA7) Program
Source: Int J Environ Res Public Health. 2022 Sep 27;19(19):12243. doi: 10.3390/ijerph191912243 (PMC9566157; doi:10.3390/ijerph191912243)
Supplement: Supplementary file 1 [file ijerph-19-12243-s001.zip › ijerph-1846094-supplementary.pdf]

**Supplementary File 1**

**Example PICHA7 Voice Mobile Health (mHealth) Message:**

*Hello! My name is Dr. Picha from Bukavu Provincial Hospital. My team has visited your family recently to share with you the information about cholera. During the next three months, I will be calling you weekly to remind you how to protect your family from cholera and other severe diarrheal diseases. Our calls and messages are free.*

*Right now, and for the next 7 days, your family is at very high risk for getting severe diarrhea again. Please use the handwashing station and safe water storage bucket that we provided to help prevent this. Have you set up the blue safe water storage bucket on the blue stool? Please also add the chlorine tablet and cover it with the lid and wait 30 minutes before drinking. Also make sure the handwashing station is sitting on the red stool provided, with the soapy water bottle or bar soap and a clean cloth next to it. Be sure to share this message with your family! I'll talk to you again. Take care!*

**Summary SMS of PICHA7 Voice mHealth Message**

*For the next 7 days, your family is at very high risk for getting severe diarrhea again. Put your handwashing station on the stool provided and add chlorine tablets to the safe water storage container. Share the message. -Dr. Picha*

**Example PICHA7 Interactive Voice Response (IVR) mHealth Message**

*Hello, this is Dr. Picha on the phone from the Bukavu General Hospital, Congratulations for completing the 7-day high risk period. It is important to remember that your household members are still vulnerable for cholera and severe diarrheal diseases. It is important you always treat*

*your drinking water. The chlorine tablets may have run out by now, but that is okay. Boil your drinking water.*

*I am here with Mwanza, the mother of patient that came to the CTC for treatment. She has forgotten the correct way to boil her water and has asked for your help. Please help her. You can answer by pressing the 1 or 2 button on your phone. Your answer will be free.*

*What is the correct way to boil your drinking water?*

***Option 1:*** *If you need to boil your water for 1 hour for it to be safe press the 1 button on your phone.*

***Option 2:*** *If you need to boil water until large bubbles rise up press the 2 button on your phone.*

*You will receive the correct answer after you press 1 or 2. The answer to this is free.*

***Pressed 1****: Nice try. Boiling water safely is easy. You only have to boil water until large bubbles rise up, a rolling boil. After boiling, please put your water in the blue drinking water vessel we provided with the lid on. Only use the tap to dispense water. Share this message.*

***Pressed 2:*** *Correct. Boiling water safely is easy. You only have to boil water until large bubbles rise up, a rolling boil. After boiling, please put your water in the blue drinking water vessel we provided with the lid on. Only use the tap to dispense water. Share this message.*

**Summary PICHA7 SMS for IVR mHealth Message**

*Staying healthy is lifelong. Your chlorine may be gone. It's okay. Boil your water. It's easy. Heat water until large bubbles rise up then put it in the blue bucket with the lid on. Share the message.*

*-Dr. Picha*
